# A metal ion–dependent conformational switch modulates activity of the *Plasmodium* M17 aminopeptidase

**DOI:** 10.1016/j.jbc.2022.102119

**Published:** 2022-06-09

**Authors:** Chaille T. Webb, Wei Yang, Blake T. Riley, Brooke K. Hayes, Komagal Kannan Sivaraman, Tess R. Malcolm, Stephen Harrop, Sarah C. Atkinson, Itamar Kass, Ashley M. Buckle, Nyssa Drinkwater, Sheena McGowan

**Affiliations:** 1 Biomedicine Discovery Institute, Department of Microbiology, Monash University, Clayton Melbourne, VIC, Australia; 2 Biomedicine Discovery Institute, Department of Biochemistry and Molecular Biology, Monash University, Clayton Melbourne, VIC, Australia; 3 Australian Synchrotron, ANSTO, Clayton, VIC, Australia; 4 Victorian Life Sciences Computation Center, Monash University, Clayton, VIC, Australia

**Keywords:** metalloprotease, metalloenzyme, enzyme, oligomer, cooperative, molecular dynamics, X-ray crystal structure, biochemistry, protein dynamics, DHDPS, dihydrodipicolinate synthase, LAP, leucyl aminopeptidase, MD, molecular dynamics, PC, principal component, PCA, principal component analysis, *Pf*A-M17, M17 aminopeptidase from *Plasmodium falciparum*, Pol-η, polymerase-η, *Pv*-M17, M17 aminopeptidase from *Plasmodium vivax*, RMSF, root mean square fluctuation

## Abstract

The metal-dependent M17 aminopeptidases are conserved throughout all kingdoms of life. This large enzyme family is characterized by a conserved binuclear metal center and a distinctive homohexameric arrangement. Recently, we showed that hexamer formation in *Plasmodium* M17 aminopeptidases was controlled by the metal ion environment, although the functional necessity for hexamer formation is still unclear. To further understand the mechanistic role of the hexameric assembly, here we undertook an investigation of the structure and dynamics of the M17 aminopeptidase from *Plasmodium falciparum*, *Pf*A-M17. We describe a novel structure of *Pf*A-M17, which shows that the active sites of each trimer are linked by a dynamic loop, and loop movement is coupled with a drastic rearrangement of the binuclear metal center and substrate-binding pocket, rendering the protein inactive. Molecular dynamics simulations and biochemical analyses of *Pf*A-M17 variants demonstrated that this rearrangement is inherent to *Pf*A-M17, and that the transition between the active and inactive states is metal dependent and part of a dynamic regulatory mechanism. Key to the mechanism is a remodeling of the binuclear metal center, which occurs in response to a signal from the neighboring active site and serves to moderate the rate of proteolysis under different environmental conditions. In conclusion, this work identifies a precise mechanism by which oligomerization contributes to *Pf*A-M17 function. Furthermore, it describes a novel role for metal cofactors in the regulation of enzymes, with implications for the wide range of metalloenzymes that operate *via* a two-metal ion catalytic center, including DNA processing enzymes and metalloproteases.

Intracellular proteolysis requires precise spatial and temporal control to prevent cleavage of proteins not destined for destruction. For this purpose, high-molecular weight protease enzymes are self-compartmentalized, whereby the proteolytic active sites are enclosed in inner cavities isolated from the cellular environment. Such an arrangement is often mediated by multimeric self-association, such as that seen for the family of M17 aminopeptidases, otherwise known as PepA or LAP (leucyl aminopeptidase; Clan MF, family M17) (1), which are present in all kingdoms of life. Although overall sequence conservation is low, M17 aminopeptidases possess a conserved homohexameric structure wherein a dimer of trimers encloses an inner cavity harboring the six active sites (2). The sequence and structure of the proteolytic sites themselves, as well as the reaction they catalyze, are highly conserved, all utilizing two divalent metal ion cofactors to catalyze the removal of select N-terminal amino acids from short peptide chains (2). This proteolysis reaction contributes to intracellular protein turnover, a fundamental housekeeping process across all living organisms (3).

While M17 aminopeptidases are well known for their proteolytic roles, a wide range of additional functions beyond aminopeptidase activity have also been attributed to M17 family members (4). In bacteria, M17 aminopeptidases bind DNA in a sequence-specific manner to regulate transcription (5), and also form hetero-oligomers (6, 7, 8), which control site-specific DNA recombination (9, 10). In plants, M17 aminopeptidases function as part of the stress response (11) that, in addition to aminopeptidase activity (12), has been suggested to result from molecular chaperone activity (13). Furthermore, the molecular chaperone function has been attributed to a monomeric form of the enzyme, suggesting that the hexamer can dissociate into individual protomers to moderate distinct functionalities (13, 14). Therefore, although the family of M17 aminopeptidases have a highly conserved structure across different organisms, they are multifunctional, capable of performing diverse organism-specific functions far beyond peptide hydrolysis (4). These diverse functions are largely mediated by dynamic changes in macromolecular assemblies; the conserved hexameric structure clearly has an enormous capacity to moderate functionality through structural dynamics. Such dynamics are also likely to influence the mechanism of proteolysis characteristic to the enzyme family. Analysis of both the tomato M17 aminopeptidase (LAP-A1) and the M17 aminopeptidases from *Plasmodium* parasites has shown that hexamerization is required for proteolysis (15, 16) and that the metal ion environment was able to dictate the oligomeric state of *Plasmodium* enzymes (16). This finding was surprising as crystal structures of M17 aminopeptidases from a wide range of organisms show that each of the six active sites appear entirely self-contained, and the reason and mechanism by which a metal ion could modulate both hexameric assembly and subsequent proteolysis was not apparent. The hexameric assembly of M17 aminopeptidases therefore clearly plays an important role in proteolysis. Despite this, our mechanistic understanding of M17 proteolysis is based on early examinations of the bovine lens M17 aminopeptidase, which treat the six chains within the hexamer as discrete entities, and have only ever considered small-scale flexibility within a single active site (17).

Of interest to our team is the M17 aminopeptidases from *Plasmodium falciparum* (*Pf*A-M17) and *Plasmodium vivax* (*Pv-*M17), the two major causative agents of malaria in humans. *Pf*A-M17 is essential for the blood stage of the parasite life cycle (18), and inhibition of *Pf*A-M17 results in parasite death both *in vitro* (19) and *in vivo* (20). Consequently, the *Plasmodium* M17 enzymes are exciting targets for the development of novel antimalarials (20, 21, 22, 23, 24, 25). The crystal structure of both *Pf*A-M17 and *Pv*-M17 shows the homohexameric arrangement characteristic of M17 aminopeptidase enzymes (Fig. 1*A*), with the six active sites orientated inward and accessible to the central cavity (Fig. 1, *B* and *C*) (26). Each active site appears independent and catalytically competent—all the apparent machinery to achieve proteolysis can be found in each site with not obvious relationship to other members of the hexameric assembly. Therefore, we were curious to understand how the metal ions mediate the formation and/or stabilization of protein–protein interactions between M17 subunits.

**Figure 1:**
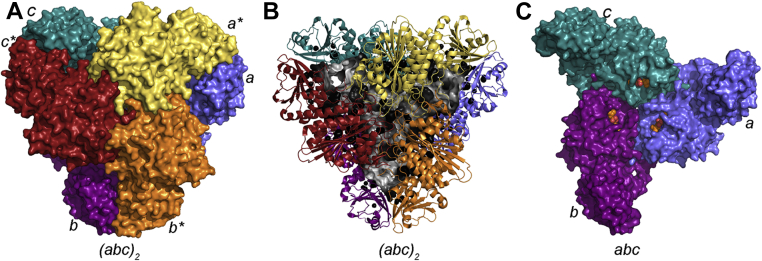
**The crystal structure of *Pf*A-M17.***A*, *Pf*A-M17 is a homohexamer composed of a dimer of trimers. Three chains, denoted A (*blue*), B (*purple*), and C (*teal*) interact through their C-terminal domains to form a “trimer,” which interacts with an identical trimer made up of D (*yellow*), E (*red*), and F (*orange*) to form the hexamer. *B*, *Pf*A-M17 (*cartoon*) possesses a large internal cavity (*gray surface representation*) that contains the six active sites. *C*, a single trimeric face (ABC) of the hexamer, shown in the same orientation as A (front trimeric face hidden). *Orange spheres* show binding of substrate-analog bestatin and indicate the active sites, which are exposed to the inner cavity. (Protein Data Bank ID: for *Pf*A-M17, 3KQZ) (26). *Pf*A-M17, M17 aminopeptidase from *Plasmodium falciparum*.

Here, we have examined the role hexamerization plays in the *Pf*A-M17 mechanism through simulations, X-ray crystallography, and mutational analyses. With this comprehensive strategy, we characterized the range of protein motions inherent to *Pf*A-M17 and probed the specific contribution of those motions to enzyme function. Based on the results, we propose a novel model for how *Pf*A-M17 functions at an atomic level. In the interior of the catalytic cavity, six dynamic regulatory loops (denoted L13), one contributed from each protomer, function cooperatively to mediate communication between the subunits of the hexamer. This loop motion shuttles a key conserved lysine residue, Lys386, between neighboring active sites and promotes rearrangement of the binuclear metal center. Based on mutagenesis and biochemical analyses, we propose a novel role for Lys386 in stabilization of the hexameric assembly through second-shell coordination interactions with the binuclear center, as well as shuttle of the substrate/product in/out of the active site. These roles for Lys386 are in addition to the previously proposed role of catalytic base in the reaction mechanism and stabilization of the substrate throughout the reaction. Furthermore, we show that the L13 loop motion coupled with rearrangement of the binuclear metal center effects a switch between active and inactive enzyme states, thereby acting as a dynamic regulatory mechanism. Based on the evidence, we propose that this metal-dependent switch is an inherent component of the *Pf*A-M17 catalytic cycle and is the key physiological mechanism that may regulate cellular proteolysis under different environmental conditions.

## Results

### Hexameric assembly stabilizes and links the *Pf*A-M17 active sites

Previously, we showed that monomeric *Pf*A-M17 is catalytically inactive despite having what appeared to be an intact active site ready for proteolysis (16). To try and understand why the monomeric form of *Pf*A-M17 is inactive, we turned to all-atom MD simulations to compare the motions of the inactive monomer to the active hexamer. We performed all-atom (protein + zinc + water) MD simulations of both arrangements in triplicate (monomer, 3 × 200 ns; hexamer, 3 × 400 ns). Analysis of the RMSD of the Cα atoms over the course of the simulations indicated that the monomer underwent greater structural changes than the hexameric enzyme (average RMSD of monomer = 4.1 ± 0.5 Å, Supporting information 1A; average RMSD of hexamer = 2.5 ± 0.04 Å, Supporting information 1B). Furthermore, in the simulation of monomeric *Pf*A-M17, variation was observed between triplicate runs (Supporting information 1A). This is in contrast to the hexamer simulation where all three runs showed a similar RMSD profile (Supporting information 1B). This clear difference in the dynamics of hexameric *versus* monomeric *Pf*A-M17 suggests that the hexameric assembly may act to stabilize the monomer.

To determine how the stability of the hexamer translates to the catalytic machinery, we examined the local environment of the active sites throughout the simulation. The catalytic reaction mechanism of M17 aminopeptidases relies on the deprotonation of a catalytic water or hydroxyl ion for nucleophilic attack on the peptide carbonyl carbon (17, 27). In the simulation of monomeric *Pf*A-M17, we did not observe a stable position for a water molecule within the active site (Supporting information 2A and C). In contrast, in the simulation of hexameric *Pf*A-M17, we observed a stable water conformation wherein it associates with the site 1 Zn^2+^ ion (Supporting information 2B and D), consistent with the position of a nucleophilic water (17, 28).

Catalysis relies on precise placement of chemical moieties within the active site, so we assessed the mobility of individual residues throughout the simulations *via* calculation of the root mean square fluctuations (RMSFs) of the Cα atoms. The RMSF analysis showed clear differences between the two simulations, particularly residues 385 to 391, which lie on a loop (referred to here as L13) flanking the active site on the interior of the central cavity. In the simulation of monomeric *Pf*A-M17, L13 exhibited a high degree of flexibility (average RMSF over three runs for L13 = 4.5 ± 0.4 Å; Supporting information 3A). However, in the simulation of hexameric *Pf*A-M17, the flexibility of the loop varied between the six chains (Supporting information 3B). In four chains (chain A, D, E, and F), L13 was relatively stable (average RMSF = 1.6–2.1 Å); however, in the remaining two chains (chain B and C), it showed a higher level of mobility (average RMSF B = 2.8 ± 0.4 Å and C = 3.1 ± 0.4 Å).

To extract the details of the motions that the hexameric *Pf*A-M17 underwent, we performed a principal component analysis (PCA) of these simulations. Projecting the trajectories on to the top two principal components (PCs) (where PC1 accounts for 62% of the total variance and PC2 10%) showed that the major motion, described by PC1, is an overall expansion of the hexamer (measured across the face of the trimer) from 120 Å to 127 Å (average of three measurements between Cα of Asn181^*a*^, Asn181^*b*^, and Asn181^*c*^). The expansion is approximately 6% of the overall hexamer size and likely results primarily from the release of crystal constraints (29). However, within PC2, we observed a substantial movement of the L13 loop, the same region identified in the RMSF analysis. In the crystal structure, and therefore the starting conformation of the MD simulation, the position of L13 is identical in all six units, where it lines the active site and extends into the solvent of the inner cavity. PCA showed that in PC2 of the *Pf*A-M17 hexamer simulation, L13 from one subunit of each trimer (B and E) moves away from its own active site, extends across the entrance of the pocket, and stretches toward the active site of the neighboring chain (Supporting information 4). In the most extreme conformation in chain E, L13 occludes the entrance to the chain E pocket and contacts L13 of the neighboring chain, F. Although L13 shows some movement in all chains within the hexamer, the extreme motion is only observed for one site per trimer (chains B and E). Interestingly, L13 contains lysine residue 386, which has a predicted role in the reaction mechanism of *Pf*A-M17 (17, 27, 30). The loop movement therefore serves not only to link neighboring active sites but also to remodel the catalytic machinery. Taken together, our MD analyses demonstrate that the multimeric assembly stabilizes the active-site environment of M17 aminopeptidases and further suggests that L13 might mediate communication between the active sites of the *Pf*A-M17 hexamer.

### Novel *Pf*A-M17 conformation captured by crystallography

Our MD simulations identified that the L13 loop is dynamic and capable of linking one *Pf*A-M17 active site to the neighboring unit within the trimer. This is the first evidence that shows the active sites of M17 aminopeptidases might be linked. However, MD simulations only provide a snapshot of the range of motion that proteins may experience, and indeed, because of the challenges of simulating metalloenzymes, may bias trajectories close to the active site (28). Therefore, we sought to validate our computational findings and demonstrate that the enzyme is physiologically capable of sampling different active-site conformations.

**Figure 2:**
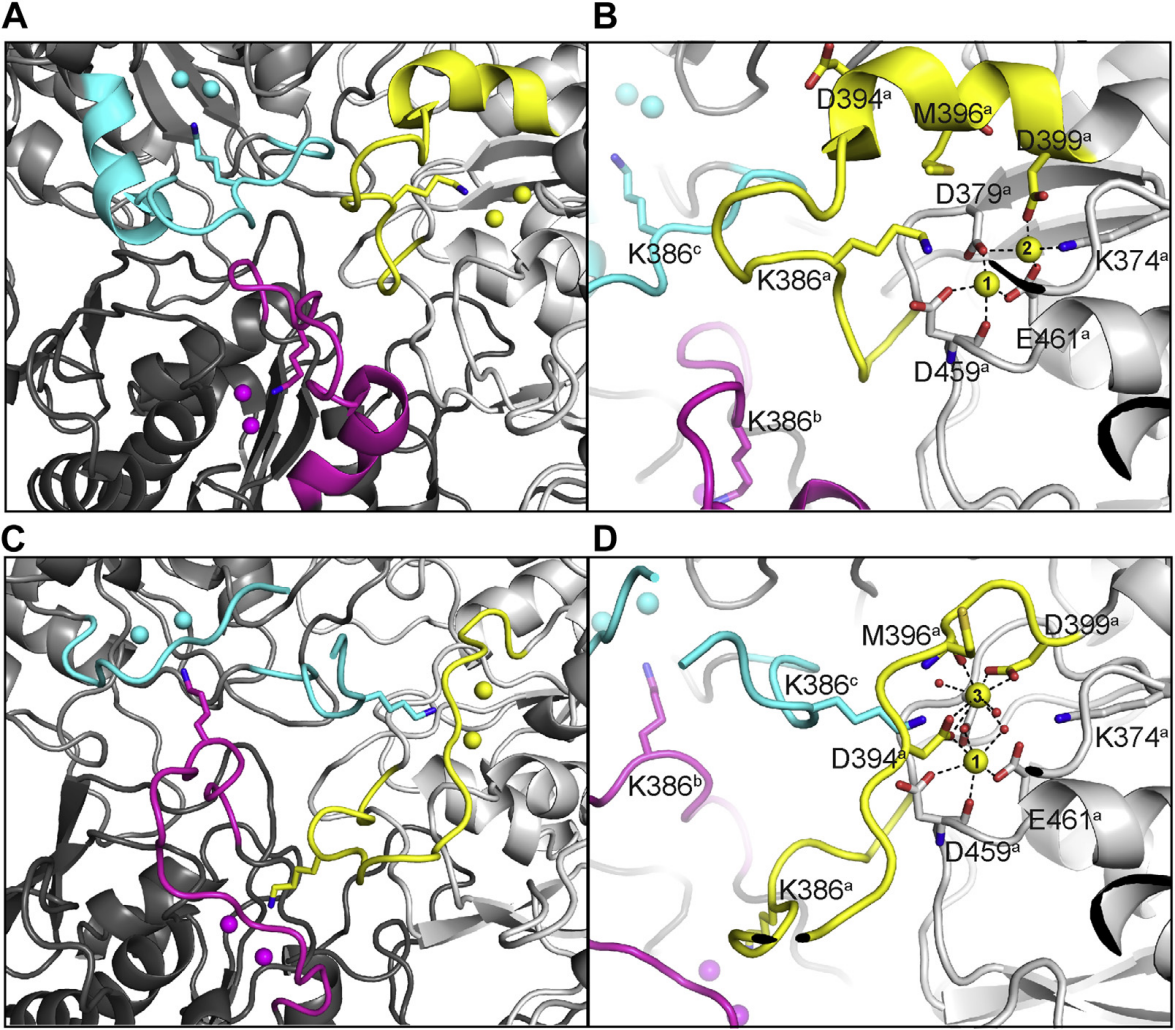
**Novel *Pf*A-M17 structure captured with the active sites linked by flexible L13 loop.***A*, active-site arrangement of *Pf*A-M17_Active_ at the junction of the ABC trimer (on the interior of the *Pf*A-M17 hexamer) showing that the active sites of each chain are distinct. Chain A is *light gray*, with L13 loop and metal ion atoms in *yellow*; chain B is *dark gray*, with L13 loop and metal ions in *magenta*, chain C is *medium gray*, with L13 loop and metal ions in *cyan*. Lys386 of all chains shown in *stick* representation. *B*, the active site of chain A in *Pf*A-M17_Active_ (Protein Data Bank [PDB] ID: 3KQZ). Orientation is similar to that in *A*. Metal-binding positions 1 and 2 are indicated, and active-site residues are shown in *stick* representation. *Subscript* denotes chain identifier. *C*, active-site arrangement of *Pf*A-M17_Inactive_ at the junction of the ABC trimer, showing that the active sites are linked by the L13 loops. Coloring and orientation consistent with *A*. Lys386 sits in the active site of the neighboring chain, effectively linking all three active sites within the trimer. *D*, active site of chain A in *Pf*A-M17_Inactive_ in same orientation as *B*. Disruption of active-site α-helix is observed, and reorganization of the active-site architecture, including metal-binding positions has occurred. Metal-binding positions 1 and 3 are indicated, and active-site residues are shown in *stick* representation (PDB ID: 7SRV). *Pf*A-M17, M17 aminopeptidase from *Plasmodium falciparum*.

**Figure 3:**
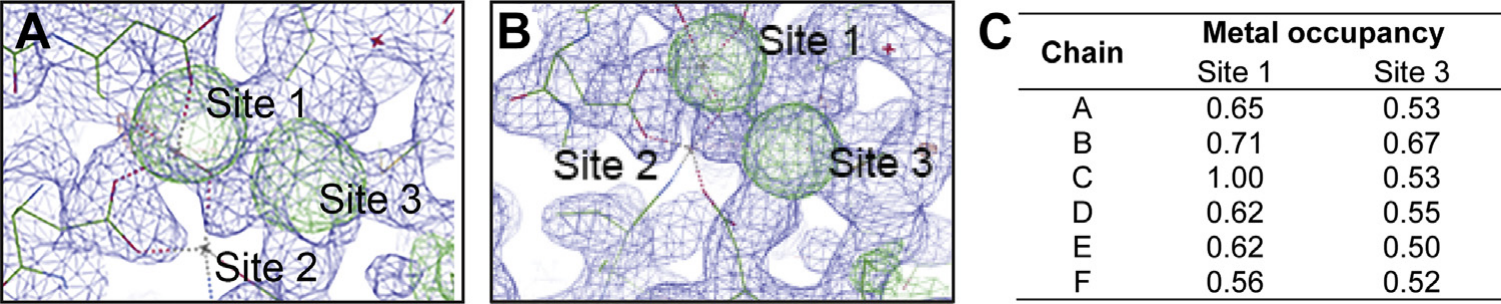
***Pf*A-M7**_**Inactive**_**crystal appears to have zinc ion density that does not fit the active conformation (Protein Data Bank [PDB] ID:****3KQZ****).***A* and *B*, electron density maps (2*F*_o_–*F*_c,_*blue*, shown at 1σ) and (*F*_o_–*F*_c,_*green*, shown at 3σ) of *Pf*A-M17_Inactive_ highlight the position of metal ion sites 1 and 3 in chain A (*A*) and chain B (*B*). The maps of *Pf*A-M17_Inactive_ are overlayed with the structure of *Pf*A-M17_Active_ (PDB ID: 3KQZ) to show the position of the original metal ions at sites 1 and 2 denoted by *gray crosses*. Site 1 metal ion clearly sits in the difference density demonstrating that site 1 is in the same position in both structures; however, the second metal ion in *Pf*A-M17_Active_ site 2 is in a distinct position to the density observed at site 3 in the *Pf*A-M17_Inactive_ structure. *C*, zinc occupancies in the *Pf*A-M17_Inactive_ structure are generally <1.0. *Pf*A-M17, M17 aminopeptidase from *Plasmodium falciparum*.

**Figure 4:**
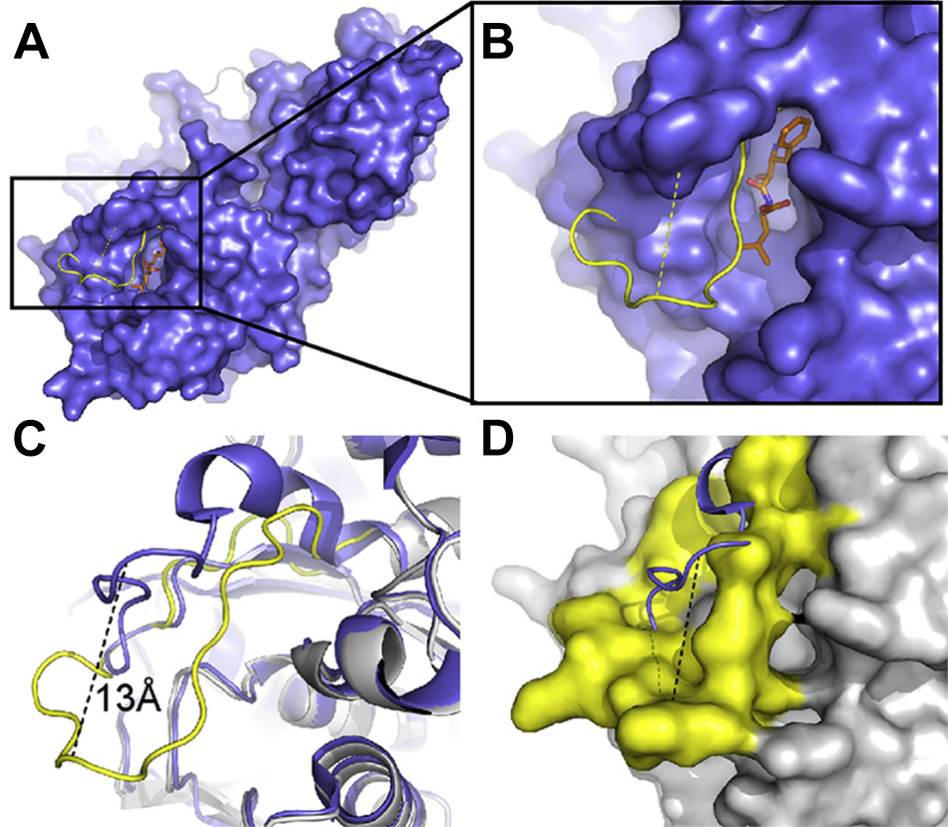
**Conformational change from active to inactive *Pf*A-M17 mediated by flexible L13 loop.***A* and *B*, overlay of a single representative monomer (chain A) of the “inactive” *Pf*A-M17 conformation (*Pf*A-M17_Inactive_, *yellow cartoon*) with “active” *Pf*A-M17 (*blue surface*) in complex with substrate-analog bestatin (*orange sticks*). In the “active” conformation, *Pf*A-M17_Active_ (*blue*), the binding mode of bestatin (*orange*) is shown to indicate the active site. *Yellow cartoon* protruding through the surface shows the new position of the L13 loop in the “inactive” conformation of *Pf*A-M17, *Pf*A-M17_Inactive_. The loop moves ∼13 Å (*yellow dash*) to occlude the active site, occupying the same space as bestatin in the “active” structure. *C*, overlay of *Pf*A-M17_Active_ (*blue cartoon*) and *Pf*A-M17_Inactive_ (*gray and yellow cartoon*) crystal structures of *Pf*A-M17. Region and orientation is the same as that shown in *B*. *Black dashed line* indicates the ∼13 Å movement of the flexible L13 loop (*yellow*), which disrupts an active-site alpha helix (*top right*, foreground) and beta sheet (*top right*, background). *D*, inverted representation of *Pf*A-M17_Inactive_ in *C*, wherein the “inactive” conformation of *Pf*A-M17 (*gray*) is shown in surface representation, with the flexible loop (*yellow*) completely occluding the active site. *Black dash* shows the 13 Å movement of the loop from the “active” conformation (*blue cartoon*). *Pf*A-M17, M17 aminopeptidase from *Plasmodium falciparum*.

We rationalized that metastable conformations of *Pf*A-M17, including variations of the position of L13, might be captured and characterized by crystallization. Therefore, we performed sparse matrix screens to identify novel *Pf*A-M17 crystallization conditions. Any conditions different to those from which the original crystal structure was optimized, diffraction data collected, and structures solved by molecular replacement. One structure, solved to 2.0 Å (Supporting information 5), showed a previously unobserved conformation. Overall, the quaternary structure is similar to previous *Pf*A-M17 structures with the hexamer arranged as a dimer of trimers and the domain arrangement in each unit consistent with previous conformations. However, the new structure shows a vastly different active-site arrangement to any previously published M17 structure (Fig. 2 and Supporting information 6 show a morph between the two alternate structures). In the previously determined, original *Pf*A-M17 structure in the “active” conformation, henceforth referred to *Pf*A-M17_Active_, L13 lines the active site and extends into the solvent of the inner cavity (Fig. 2, *A* and *B*). In the new conformation, L13 loops of each of the six chains cross the entrance to the active sites and extend to the active site of the neighboring chain in the trimer (Fig. 2, *C* and *D*). This rearrangement is remarkably similar to those observed for chains B and E of the MD simulations. In this new position, the six key Lys386 residues each occupy the active site of the neighboring chain (Figs. 4*C* and 3*D*). The L13 rearrangement therefore results in a direct link between the three active sites of each of the trimers within hexameric *Pf*A-M17. A relatively large movement of approximately 13 Å is required to elicit the conformational changes in each of the six loops, which also serves to occlude the entrances to the six sites (Fig. 4). To obtain the slack within L13 to adopt this extended occluded conformation, the secondary structure at both ends of the loop has been disrupted (Fig. 2*A* compared with Figs. 2*C* and 4*C*). This includes disruption of the α-helical structure of 10 residues (392–401), which results in substantial shortening of an active-site helix (392–417), and complete disruption of a short β-strand (residues 372–379).

The active-site rearrangement extends beyond protein backbone changes and appears to include the two catalytic metal ions (Supporting information 6). Based on kinetic and biophysical characterization, the two metal sites of the M17 aminopeptidases have previously been termed site 1 and site 2, whereby site 1 is that closest to the mouth of the active site (Fig. 2*B*) (2). Previous crystallographic studies have shown that site 1 metal ions are readily lost, whereas site 2 is always occupied (most often by Zn^2+^ or Mn^2+^ (4)), and kinetic studies have suggested that removal of site 2 metal ion results in ablation of catalytic activity (31). Therefore, site 2 is generally considered the tight-binding catalytic site and site 1 the weak-binding regulatory site (26, 31, 32, 33). Remarkably, in the novel conformation described here, we observed rearrangement of the metal-binding positions. Site 1, as expected, appears occupied with a Zn^2+^ ion in a capped trigonal bipyramidal coordination geometry, but refinement suggests that the position is not fully occupied by a Zn^2+^ ion (∼0.6–1.0 occupancy) (Fig. 3). The interesting finding is that the “catalytic” metal in site 2 is absent (Fig. 2*D*) and further, there is electron density indicative of a metal ion observed in a third, previously uncharacterized site (Fig. 3). This position, which we now refer to as site 3, is 3.8 Å from site 1 and coordinated by the side chains of Asp394 and Asp399, the main chain oxygen of Met396, and two ordered water molecules (Fig. 2*D*). Based on our previous experience of zinc being present in M17 crystals, we modeled a Zn^2+^ ion into the density, but this ion could also only be satisfactorily refined with partial occupancy in all six chains (∼0.5–0.7 occupancy, Fig. 3*C*). Site 3 is only available when L13 has undergone the aforedescribed conformational change; the structure therefore shows a completely remodeled active site, including the architecture of the binding pocket and the catalytic binuclear center. This novel active-site conformation is incompatible with binding substrate, so as a result, was termed *Pf*A-M17_Inactive_ with an “inactive” conformation.

### *In situ* crystalline protein dynamics observed in *Pf*A*-*M17

Typically, crystal structures of recombinant *Pf*A*-*M17 contain zinc ions in their active sites, presumably scavenged during purification (26). However, the occupancy of the metal ions within the active site of *Pf*A-M17 can vary, and our *Pf*A-M17 crystals are routinely soaked to promote full occupancy of the metal ion positions (21, 22, 26, 34). The *Pf*A-M17_Inactive_ crystal was not soaked in Zn^2+^ prior to data collection; however, we presumed that if metals were present, they would likely be zinc ions. Therefore, to confirm the metal positions and metal identity within the new *Pf*A-M17_Inactive_ structure, anomalous data were collected at the zinc edge. However, despite our best efforts, we were unable to collect enough data from the crystal to obtain a strong anomalous signal, likely because of variable occupancy and a poor ratio of zinc ions to protein atoms (12: ∼23,000) and water (∼1800 waters).

**Figure 5:**
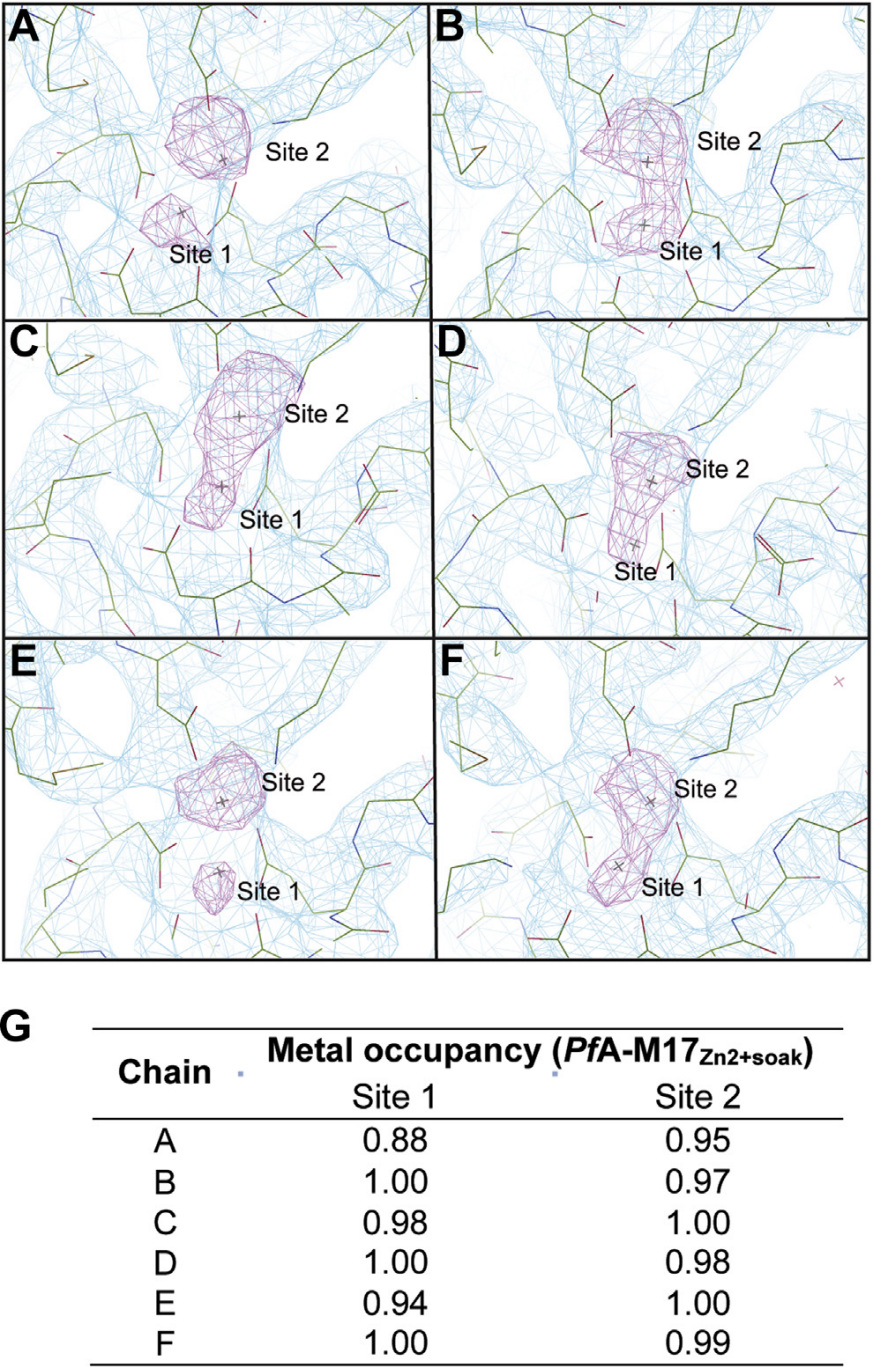
**Anomalous density confirms the presence of two zinc atoms in the active site of *Pf*A-M17.***A*–*F*, the density map (2*F*_o_–*F*_c_, *light blue*) from *Pf*A-M17_Zn2+soak_ at one σ with the atomic structure overlayed for each chain. Anomalous density (*purple*) contoured at 4.5 σ highlights the position of zinc atoms at sites 1 and 2 of the active site, analogous to the sites observed in *Pf*A-M17_Active_. *G*, zinc ions in the *Pf*A-M17_Zn2+soak_ structure have high occupancy. *Pf*A-M17, M17 aminopeptidase from *Plasmodium falciparum*.

To overcome the poor signal, we reasoned that by soaking crystals with zinc and collecting multiple datasets, a strong anomalous signal could be achieved to verify the presence and placement of each zinc atom. We produced more crystals of *Pf*A-M17_Inactive_ and soaked these crystals with a zinc solution prior to data collection. An energy scan of the soaked crystals definitively showed that only zinc was present in the crystals (Supporting information 7). Merging data from multiple datasets collected at the zinc edge successfully illuminated the anomalous signal. To our surprise, when we solved this crystal structure using molecular replacement, the position of the zinc at site 3 was absent and zinc atoms were evident in sites 1 and 2 as seen in the “active form.” Comparison of the electron density clearly shows a distinct change in the density for the zinc atoms; however, this time, the anomalous maps could confirm the presence of two zinc atoms (Fig. 5). The change in the metal coordination site between sites 2 and 3 was accompanied by a significant movement of each L13 loop, returning L13 to its active conformation that permits substrate entry to the active site (Supporting information 8; RMSD = 0.185 between the aligned L13 loops from chain A of both structures). This novel structure, named *Pf*A-M17_Zn2+soak_, reveals *in situ* evidence for the dynamic nature of L13 and the contribution of the metal ions to loop movement.

### Asp394 implicated in the formation of the catalytically competent active site in *Pf*A*-*M17

The presence of an apparent dynamic metal-binding site in *Pf*A-M17 was surprising since, to our knowledge, an equivalent site has never been observed in any M17 aminopeptidase. We were therefore curious to examine potential functional roles for the metal ion movement between sites 2 and 3. We identified Asp394 as crucial to the formation of site 3. This residue coordinates site 3 metal ion and in addition lies within the flexible L13 that has undergone substantial rearrangement (Fig. 4*D*). In contrast, in the original active conformation of *Pf*A-M17, Asp394 has no clear role; whereas close to the active site, the side chain is directed to solvent and makes no interactions (Fig. 4*B*). Therefore, to validate a physiological role for the metal ion position at site 3, we disrupted the ability of *Pf*A-M17 to coordinate metal by mutating Asp394 to alanine, *Pf*A-M17 (D394A). The effect of this mutation on enzyme activity was profound and unexpected. In comparison to *wildtype* enzyme, *Pf*A-M17 (D394A) showed greatly increased catalytic ability. The overall catalytic efficiency increased almost 10-fold, which resulted from an altered catalytic turnover rate (increased *k*_cat_, Table 1) rather than a change in *K*_*M*_ (Table 1). Given Asp394 neither does have a key structural role in the “active” conformation of *Pf*A-M17 nor in the currently accepted mechanism of hydrolysis, the change in activity observed on mutation of Asp394 could only result if the “inactive” conformation has a functional role in *Pf*A-M17 catalysis and is sampled as part of either the catalytic or regulatory mechanism.

**Table 1:** Kinetic analysis of mutant *Pf*A-M17 enzymes

Protein	[E]_tot_ (μM)	*K*_*M*_ ± SEM (μM)	*k*_cat_ × 10^−6^ ± SEM (min^−1^)	*k*_cat_*/K*_*M*_ (M^−1^ min^−1^)
*Wildtype Pf*A-M17	0.040	33.7 ± 1.5	2.5 ± 0.03	0.08
*Pf*A-M17 (D394A)	0.010	19.5 ± 1.1	11 ± 0.2	0.6
*Pf*A-M17 (K386A)	1.0	68.0 ± 7.0	0.052 ± 0.002	0.0008
*Pf*A-M17 (A387P)	0.65	45.1 ± 2.5	0.22 ± 0.004	0.005
*Pf*A-M17 (Δ388–389)	0.65	51.2 ± 5.1	0.31 ± 0.01	0.006
*Pf*A-M17 (Δ388–390)	0.080	102 ± 9	1.3 ± 0.04	0.01

### Dynamic loop regulates *Pf*A-M17 catalysis

The description of an inactive conformation of *Pf*A-M17, with active sites linked through the dynamic rearrangement of L13, has clear mechanistic implications. Lys386, within the L13 loop, has a predicted role in the *Pf*A-M17 catalytic mechanism, where it is suggested to deprotonate the catalytic water and position the peptide substrate for catalysis. However, it is also a key player in the rearrangement of L13 (Fig. 4*B* *versus* 4*D*). In the inactive conformation, Lys386 of each chain occupies the active site of the neighboring chain. To probe the importance of Lys386 to the function of *Pf*A-M17, we mutated it to an alanine. *Pf*A-M17 (K386A) showed substantially reduced activity compared with the *wildtype* enzyme (Table 1). Determination of catalytic parameters showed that this retardation of activity is largely because of not only a decrease in the ability of the enzyme to accelerate the reaction (measured by a decrease in *k*_cat_; Table 1) but also a slight change in *K*_*M*_ (twofold increased compared with *wildtype*; Table 1). While the disrupted activity demonstrates that Lys386 is key to *Pf*A-M17 function, it does not discriminate between a direct role in the catalytic reaction *versus* structural roles. To investigate further, we examined the effect of the K386A mutation on the solution state of *Pf*A-M17 using analytical gel filtration. The *wildtype* enzyme elutes as a large molecular weight species with predicted molecular weight of 330 kDa (Supporting information 9), consistent with the calculated molecular weight of a hexamer (350 kDa). In contrast, *Pf*A-M17 (K386A) elutes as a smaller species with predicted molecular weight of 145 kDa (Supporting information 9). To assess the protein stability of *Pf*A-M17 (K386A), we used differential scanning fluorimetry to show the mutant exhibits a similar melting temperature to the *wildtype* enzyme (Supporting information 9). Lys386 therefore plays a key role beyond catalysis that influences the stability of the hexameric assembly that is essential for proteolysis.

To determine if Lys386 alone stabilizes the hexamer, or alternatively, if it is the motion of L13 shuttling Lys386 between neighboring protomers, we created a series of L13 variants. We first reduced the mobility of L13 by introducing a proline in place of Ala387, a loop residue that does not have any binding interactions in either the active or inactive conformations of *Pf*A-M17. Similarly to *Pf*A-M17 (K386A), *Pf*A-M17 (A387P) showed substantially reduced catalytic activity (Table 1). Large concentrations of enzyme were required to measure enzyme activity, which manifested as reduced product turnover (decreased *k*_cat_) compared with the *wildtype* enzyme. However, unlike *Pf*A-M17 (K386A), the hexameric assembly of *Pf*A-M17 (A387P) is intact, as measured by analytical size-exclusion chromatography (Supporting information 9). Therefore, the ablation of activity is likely because of a decrease in loop flexibility, not an alteration in oligomerization.

### Regulatory loop differs throughout M17 aminopeptidase family

M17 aminopeptidases play vital roles in a wide range of physiological processes throughout all kingdoms of life. We therefore sought to determine whether the M17 enzymes from other organisms might operate *via* similar mechanisms to what we have determined for *Pf*A-M17. We compared the *Pf*A-M17 aminopeptidase sequence to that of the other human *Plasmodium* pathogens as well as the key rodent malaria model species (Supporting information 10A). The results show that the L13 residues in the *Plasmodium* enzymes are highly conserved, with the metal binding Asp394 residue (numbering as per *Pf*A-M17) present and L13 loop length and composition highly similar (only one enzyme showing a single residue change). These results suggest that within the *Plasmodium* sp, the M17 aminopeptidases may employ a similar use of metal ions to promote proteolysis.

Looking beyond the *Plasmodium* family members, we investigated the M17 aminopeptidases from the curated and annotated MEROPS clan MF, family M17.001 (35). We used the provided sequence alignment of 140 M17 aminopeptidases from a range of different organisms and compared the region of the regulatory loop (Supporting information 10B and C). The alignment showed that while Lys386 and Asp399 (involved in coordination of metal site 2 and 3) are completely conserved throughout the M17 aminopeptidase family, neither the regulatory loop following it nor the aspartic acid residue that links the regulatory loop with the third metal-binding site are conserved. Closer examination of the frequency of each type of amino acid found in our third metal-binding position (shown as 394 numbering as per *Pf*A-M17) shows that most members have either an acidic (Asp/Glu) or hydrophobic residue (Pro/Ala/Val). Based on the results of our own mutational analyses, we expect enzymes with an acidic residue would be capable of coordinating the third metal, however, the latter group likely incapable of the loop dynamics as we described for *Pf*A-M17. The sequence analysis therefore suggests that there may be two distinct mechanisms of proteolytic regulation within the family of M17 aminopeptidases.

## Discussion

Our appreciation of protein flexibility and dynamics has grown remarkably from the early static lock-and-key model, to our current understanding that proteins are dynamic entities capable of extreme flexibility. For enzymes, flexibility influences substrate binding (36, 37) and reaction mechanism (38, 39), allowing precise control of reactions that if left unregulated, could be harmful to the cell. Despite the clear importance of enzyme flexibility, there are few cases where dynamics, and how those dynamics link to function, are truly understood on an atomic level.

M17 aminopeptidases were first characterized as homohexamers during early examinations of the bovine lens M17 aminopeptidase (40). The conserved arrangement is now one of the most distinctive features of the family, along with the arrangement of the binuclear metal center (4). It is therefore somewhat surprising that almost 50 years on, we understand neither the contribution the quaternary structure makes to enzyme function nor how the metal ion identity or occupancy dictates quaternary structure. Our laboratory has an interest in the *Plasmodium* M17 aminopeptidases as novel drug targets for new antimalarials (22, 25, 34). Our investigation into the structure, function, and inhibition of the enzymes as drug targets have led to our recent studies showing that both metal ion abundance controls the oligomeric equilibrium of the enzyme, which in turn regulates proteolysis (16, 34).

The actual mechanism by which the metals were able to control the assembly (and disassembly) of the hexamer remained elusive. To answer this question, we probed the protein dynamics. It was clear from our previous study that the monomeric subunit of M17 was inactive regardless of its metal ion environment and that the hexamer was the active species that was formed in the presence of metal ions. Our analyses of the MD simulations of inactive monomeric enzyme compared with active hexameric enzyme suggest that hexamerization enables catalysis by stabilizing the protomers, thus shielding the essential nucleophilic water. Such a mechanism of preserving active-site stability by oligomerization has been previously described, for example, dihydrodipicolinate synthase (DHDPS), which utilizes a tetrameric arrangement to stabilize the catalytic dimerization interface for optimal catalytic efficiency (41). Furthermore, formation of the DHDPS tetramer is promoted by substrate binding (42), thus, similarly to *Pf*A-M17, DHDPS demonstrates a complex interplay between substrate binding, oligomerization, and catalysis that is mediated by protein dynamics.

Beyond a role in active-site stability, the hexameric arrangement of *Pf*A-M17 also allows communication between neighboring active sites through the movement of a dynamic regulatory loop. While first identified in the MD simulations of the hexameric enzyme, we were able to validate the motion using X-ray crystallography, whereby we captured an inactive conformation of the enzyme. In this conformation, the three active sites of each trimer are linked by regulatory loops (L13 loops, one contributed from each protomer), and the binuclear metal center has undergone structural rearrangement. Our mutational analysis of the regulatory loop and metal site 3 demonstrated that loop movement is intrinsically coupled with rearrangement of the binuclear metal center and that these metalloprotein dynamics are an inherent component of *Pf*A-M17 catalytic function. This does not appear to be a result of changes to the hexameric assembly but rather the ability of the regulatory loop to mediate communication between the active sites. The inactive conformation was metal dependent, as when we soaked the crystals in zinc, we changed the affinity of the metal sites and the protein returned to an active conformation, with both the metals and L13 in a conformation ready for hydrolysis. Based on the biochemical and structural results, we propose a model in which the transition between the active and inactive conformations of *Pf*A-M17 is part of a dynamic regulatory mechanism that has evolved to moderate the rate of aminopeptidase activity under different environmental conditions.

To fully characterize how the dynamic regulatory loop moderates catalysis, the role of the highly conserved active-site lysine residue (Lys386), which is shuttled between the active sites of neighboring chains, must be considered. This residue has previously been proposed not only to stabilize the substrate in the active site but also to act as the catalytic base in the reaction mechanism (17, 27, 30). However, here, we demonstrated that the function of Lys386 extends beyond catalysis and includes a role in hexamer stabilization. In our previous study and our MD simulations reported here, we have shown that metal ions affect hexamerization and hexamerization influences active-site stability. Therefore, it is feasible that the inverse is also true, and that loss of Lys386 affects hexamerization *via* destabilization of the active site. Genna *et al.* (43) reported a comprehensive study comparing the structures and active-site dynamics of a diverse range of DNA and RNA processing enzymes that function *via* a binuclear metal center. The analysis resulted in the identification of a structurally conserved active-site architecture wherein basic amino acids occupy conserved positions in relation to the binuclear metal center and interact with the metals through the second coordination shell (43). Furthermore, the structurally conserved residues interact with substrate, despite hailing from a diverse range of enzyme families. Importantly, other enzymes with functions beyond DNA processing have described similar architecture, including the hexameric oxylate decarboxylase (44). The parallels between the architecture described by Genna *et al.* (43) and those for the M17 aminopeptidases are striking. The distance between Lys386 of *Pf*A-M17 and the metal center in both the active and inactive conformations (∼4 Å) is consistent with occupation of the second shell of metal ions, and Lys386 likely interacts with substrate. How then, does mutation of Lys386 cause dissociation of the hexamer? In the *Plasmodium* M17s as well as the tomato LAP-A1, mutation of the metal-coordination residues as well as the conserved lysine results in hexamer destabilization (34, 45). Furthermore, our structural characterization of the nonproteolytically active tetrameric intermediate of *Pv*M17 identified that in the absence of metal ions, the active site and the L13 loop are disordered (34). Therefore, we propose that Lys386 stabilizes the binuclear center through interactions with the second coordination shell, and that in its absence, in *Pf*A-M17 (K386A), the binuclear center and active site are destabilized, which in turn disrupts hexamerization.

Further parallels can also be made between the *Pf*A-M17 system and the diverse enzymes that operate *via* the conserved two-metal ion mechanism (with associated basic residue). A particularly striking similarity is observed between the human DNA polymerase-η (Pol-η) and *Pf*A-M17; Pol-η operates *via* a highly dynamic and cooperative process that involves transient recruitment of a third metal ion (46). The conserved basic residue of Pol-η, Arg61, is highly flexible, and specific conformations of Arg61 (sampled as part of a large equilibrium ensemble) serve to recruit incoming substrate, facilitate the reaction, and act as an exit shuttle (46). Such a role could readily be envisaged for Lys386. This is particularly feasible given the flexibility of the loop region (suitable for guiding substrate entry and product exit) and the proposed role in substrate positioning (for reaction facilitation).

Given that the diverse family of M17 aminopeptidases operate within a wide range of different organisms, it is entirely reasonable to expect that the enzymes have evolved to operate differently within diverse environments. Indeed, it has been suggested that flexible loops in proteins can facilitate the emergence of novel functions, which results in divergent evolution (47, 48, 49). In terms of *Plasmodium* parasites, the concentrations of different divalent metal cations are known to fluctuate throughout the life cycle (50). Therefore, the metal-dependent switch described here could potentially serve to moderate the rate of proteolysis to suit different metabolic demands throughout the complex parasite life cycle (13, 14).

The implication of the two-metal center rearrangement, which includes a third metal-binding site, should also be considered further. The metal sites of M17 aminopeptidases are most often occupied by zinc ions (Zn^2+^); however, can also be exchanged for other metals, such as cobalt (Co^2+^), manganese (Mn^2+^), magnesium (Mg^2+^), and copper (Cu^2+^) (4). Previous examinations have focused on assigning identity and role to each of the site 1 and site 2 metal ions, with each of them considered to have distinct catalytic roles (regulatory *versus* catalytic). It has never before been considered that the metal positions themselves might change. The finding that the binuclear site is dynamic, and that site 3 metal ion plays a role in the catalytic cycle of *Pf*A-M17, therefore carries substantial implications for previous and future work. In crystal structures, M17 aminopeptidases have often been observed with only a single metal ion bound. These one-metal ion forms of the enzyme have been used to interpret kinetic examinations; biphasic curves that result from titration of metal chelators into *Pf*A-M17 have been interpreted as two-metal ion bound (100% activity), one-metal ion bound (∼50% activity), and unbound (irreversibly inactivated) forms of the enzyme (31). However, our characterization of a third metal-binding site, which is sampled as part of the catalytic reaction, casts doubt on that interpretation. In a separate line of study, we have recently characterized a novel mode of *Pf*A-M17 inhibition, whereby small-molecule inhibitors reversibly displace the site 2 “catalytic” metal (25). Furthermore, studies of a λ-exonuclease, which possesses the conserved architecture discussed previously (two-metal ion mechanism with associated basic residue), proposed transient binding of a third metal ion, and in addition, demonstrated that the timing of both coordination and dissociation of each of the binuclear metal ions is important to the catalytic cycle (51). Therefore, *Pf*A-M17 could potentially transition between a monometal, bimetal, and trimetal ion center as part of the catalytic cycle.

Taking all this into account, we propose a novel mechanism for the regulation of *Pf*A-M17 activity through interplay of metalloprotein regulatory dynamics. We suggest that *Pf*A-M17 exists in a dynamic equilibrium between an inactive conformation, which does neither possess the active-site arrangement to accept substrate nor perform catalysis, and an active conformation, capable of product turnover. Transition between these conformations is mediated by the cooperative movement of six regulatory loops (one contributed from each unit of the hexamer), which, through repositioning Lys386, mediate rearrangement of the binuclear metal center. Finally, we propose this transition occurs in response to specific environmental signals, which act to regulate the rate of *Pf*A-M17 catalysis according to physiological demands of the *Plasmodium* parasite. Understanding both the physiological metal identity and concentration will be critical to understanding how metal ions influence the biological function of *Pf*A-M17. Like many metalloaminopeptidases, *Pf*A-M17 displays differential behavior dependent on metal identity and concentration, which we now understand to be a result of the profound influence of the metals on active-site conformation and in turn on the formation and stability of hexamer. We routinely use recombinant *Plasmodium* M17 as it can be readily purified in sufficient quantity for the type of detailed work outlined here. Whilst natively purified protein would provide more insight into the biological implications of our findings, our attempts to purify *Pf*A-M17 from parasites with sufficient yield have not been successful. A greater understanding of the physiological metal environment that the M17 aminopeptidases experience would allow us to connect what we know from our recombinant studies to how the mechanism is utilized to achieve the desired biological outcome.

## Conclusion

The M17 aminopeptidases have been of interest as key players in a wide range of physiological processes for over 50 years. From a protein mechanics perspective, the conserved hexameric assembly, containing six discrete active sites with binuclear metal centers, represents an exciting system in which to investigate the role of dynamic cooperativity and function within a large oligomer. Herein, we have shown that the hexameric assembly is absolutely essential for the proteolytic activity of *Pf*A-M17. The arrangement stabilizes and preserves key catalytic machinery within the six active sites and further plays a role in the regulation of aminopeptidase activity. Within the hexamer, each of the two disc-like trimers operate independently; a flexible loop links each of the three active sites within each trimer to its neighbor, thereby operating cooperatively to convert the enzyme from the inactive to active state. Furthermore, movement of the regulatory loop is coupled with a rearrangement of the binuclear metal center. Our studies show that not only do metal ion dynamics exist within the active site but also the positions of the metals are manipulated to control the activity of the enzyme. We therefore propose that the dynamic transition between inactive and active states is part of a key regulatory mechanism that controls the activity of *Pf*A-M17 and that the transition is moderated by changes in the physiological environment.

## Experimental procedures

### Molecular biology, protein production, and purification

The cloning of the truncated *Pf*A-M17 gene (encoding amino acids 85–605) into pTrcHis2B has been previously reported (26). Site-directed mutagenesis was performed by PCR and confirmed by DNA sequencing, while deletion mutants were synthesized and subcloned into pET-21d(+) (GenScript). *Escherichia coli* strains DH5α and K-12 were used for DNA manipulation, and BL21(DE3) was used for protein expression. Recombinant His_6_-tagged *wildtype* and mutant *Pf*A-M17 were expressed using an autoinduction method as previously described for *wildtype Pf*A-M17 (26). Proteins were expressed and purified as has previously been described for *wildtype Pf*A-M17 (26) using a two-step purification procedure of nickel–nitrilotriacetic acid agarose column followed by gel filtration chromatography on a Superdex S200 10/300 column in 50 mM Hepes (pH 8.0) and 150 mM NaCl buffer. Analytical gel filtration chromatography was performed using 100 μg of *wildtype* and mutant *Pf*A-M17 purified protein on a Superdex S200 Increase 10/300 equilibrated in 50 mM Hepes (pH 8.0), 300 mM NaCl, and 1 mM MnCl_2_ buffer. Approximate molecular weight of eluate was calculated by interpolation of a standard curve constructed with appropriate molecular weight standards (Gel Filtration Calibration Kit HMW; GE Healthcare). Nanoscale differential scanning fluorimetry was carried out in a Prometheus NT.48 instrument from NanoTemper Technologies with an excitation wavelength of 280 nm. Capillaries were filled with 15 μl of *Pf*A-M17 proteins (1 mg/ml in size-exclusion chromatography buffer; 50 mM Hepes [pH 8.0], and 150 mM NaCl), placed into the sample holder, and the temperature was increased from 20 to 95 °C at a ramp rate of 1 °C/min. Each protein was tested in triplicate. All experiments were performed at the Monash Molecular Crystallization Facility.

### Aminopeptidase assays and analysis

Aminopeptidase activity was determined by measuring the release of the fluorogenic-leaving group, l-leucine-7-amido-4-methylcoumarin hydrochloride (Sigma–Aldrich; catalog no.: L2145) (H-Leu-NH-Mec), from the fluorogenic peptide substrate H-Leu-NHMec as described previously (52). Briefly, assays were performed in triplicate and carried out in 50 μl total volume in 100 mM Tris–HCl, pH 8.0, 1 mM MnCl_2_ at 37 °C, and activity was monitored until steady state was achieved. Fluorescence was measured using a FluoroStar Optima plate reader (BMG Labtech), with excitation and emission wavelengths of 355 and 460 nm, respectively. Activity of the association mutant *Pf*A-M17 (W525A,Y533A) was assessed with 10 μM concentration of substrate H-Leu-NH-Mec and compared with *wildtype Pf*A-M17 activity assessed under identical reaction conditions.

For active enzymes, the Michaelis constant, *K*_*M*_, was calculated from the initial rates over a range of substrate concentrations (H-Leu-NH-Mec, 0.5–500 μM) with enzyme concentrations fixed at 40 nM for *wildtype Pf*A-M17, 10 nM for *Pf*A-M17 (D394A), 1000 nM for *Pf*A-M17 (K386A), 650 nM for *Pf*A-M17 (A387P), and *PfA*-M17 (Δ388–389), and *Pf*A-M17 (Δ388–390). Gain was fixed at 800 for all Michaelis–Menten assays and converted to units of product by interpolation of a standard curve constructed (fluorescence output of a range of methylcoumarin concentrations, collected at gain of 800). Kinetic parameters, including, *K*_*M*_ and *k*_cat_, were calculated with nonlinear regression protocols by using GraphPad Prism 7 (GraphPad Software, Inc).

### Crystallization, data collection, structure determination, and refinement

*Pf*A-M17 was concentrated to 10 mg/ml in 50 mM Hepes (pH 8.0) and 150 mM NaCl for crystallization. Crystals were grown by hanging drop vapor diffusion in 20% PEG3350, 0.2 M calcium acetate, with drops composed of 2 μl protein plus 1 μl precipitant. Crystals grew to large plates in 7 days. *Pf*A*-*M17 crystals were cryoprotected in mother liquor supplemented with 15% 2-methyl-2,4-pentanediol for 30 s before flash cooling in liquid nitrogen. When required, *Pf*A*-*M17 crystals were soaked in a 2 μl drop containing mother liquor with 2 mM ZnSO_4_ for 30 min at room temperature before being cryoprotected in mother liquor supplemented with 15% 2-methyl-2,4-pentanediol and 2 mM ZnSO_4_ for 30 s before flash cooling in liquid nitrogen.

All data were collected at 100 K using synchrotron radiation at the Australian Synchrotron using the microcrystallography MX2 beamline 3ID1 (53). Data were processed using XDS (54) and scaled in Aimless (55) as part of the CCP4 suite (56), or using PHENIX scale_and_merge (57). The *Pf*A-M17_Inactive_ structure was solved by molecular replacement in PHENIX Phaser (57) using the structure of unliganded *Pf*A-M17 (Research Collaboratory for Structural Bioinformatics Protein Data Bank [PDB] ID: 3KQZ) as the search model (26). Refinement was carried out with iterative rounds of model building in Coot (58, 59) and refinement using PHENIX (57). After several iterations of refinement and model building, NCS maps were disabled as an option in PHENIX, and the stereochemistry and ADP weight optimized. Water molecules and metal ions were placed manually based on the presence of *F*_o_–*F*_c_ and 2*F*_o_–*F*_c_ electron density of appropriate signal. The *Pf*A-M17_Zn2+soak_ structure was solved by molecular replacement in PHENIX Phaser (57) also using *Pf*A-M17_Inactive_ (Research Collaboratory for Structural Bioinformatics PDB ID: 7SRV) as the search model. To determine metal ion identity, X-ray diffraction data were collected at the Zn^2+^ anomalous absorption edge identified in the zinc energy scan at 1.23 Å (10,080 eV) (Fig. S8). Multiple datasets were collected, processed individually using XDS (32), and then scaled and merged using PHENIX scale_and_merge (57). The strength of the anomalous signal was confirmed by PHENIX anomalous_signal, and phases were calculated by molecular replacement using the native *Pf*A-M17_Zn2+soak_ structure (PDB ID: 7T3V). An anomalous map was calculated and set at 4.0 σ contour level to visualize density.

Both structures were validated with MolProbity (60), and figures were generated using The PyMOL Molecular Graphics System, Version 1.8.23, Schrödinger, LLC. Final structure coordinates were deposited in the PDB (IDs: 7SRV and 7T3V, respectively), and a summary of data collection and refinement statistics is provided in Supporting information 5. For clarity and consistency, the chains in the deposited structure are numbered according to the model of *Pf*A-M17_Active_ (PDB ID: 3KQZ).

### MD system setup and simulation protocol

The starting *Pf*A-M17 model for MD simulations was based on the unliganded crystal structure (PDB ID: 3KQZ) that contains two zinc ions in the active conformation. To prepare the model, missing atoms and residues (A^84, 257–261^, B^84–85, 255–262^, C^84–85, 255–259^, D^84, 255–259^, E^84–85, 152^, and F^84–85, 136, 255–261^) were rebuilt using Modeller, version 9.11 (61), protonated according to their states at pH 7.0 using the PDB2PQR server (62) and subjected to energy minimization using Modeller (61). The hexameric *Pf*A-M17 system consisted of ∼294,000 atoms with a periodic box of 167 Å × 166 Å × 121 Å, and the monomeric *Pf*A-M17 system consisted of ∼81,000 atoms with a periodic box of 120 Å × 85 Å × 94 Å (after solvation). Periodic boundary conditions were used for all simulations. System charges were neutralized with sodium counter ions. Proteins and ions were modeled using the AMBER force field FF12SB (63), the metal center was defined as described previously (28), and waters represented using the three-particle TIP3P model (64). All atom MD simulations were performed using NAMD 2.9 (developed by the Theoretical and Computational Biophysics Group in the Beckman Institute for Advanced Science and Technology at the University of Illinois at Urbana-Champaign) (65) on an IBM Blue Gene/Q cluster (monomer simulations) or x86 (hexamer simulations). Equilibration was performed in three stages. First, potential steric clashes in the initial configuration were relieved with 50,000 steps of energy minimization. Initial velocities for each system were then assigned randomly according to a Maxwell–Boltzmann distribution at 100 K. Each system was then heated to 300 K over 0.1 ns, under the isothermal–isometric ensemble (NVT) conditions, with the protein atoms (excluding hydrogens) harmonically restrained (with a force constant of 10 kcal mol^−1^ A^−2^). Following this, each system was simulated for 100 ps under the isothermal–isobaric ensemble (NPT) with heavy atoms restrained. The harmonic restraints used were reduced from 10 to 2 kcal mol^−1^ A^−2^ during the simulations. The aforementioned equilibration process was performed three times from the same starting structure in order to initiate three production simulations with different initial velocities. For production simulations, the time step was set to 2 fs, and the SHAKE algorithm was used to constrain all bonds involving hydrogen atoms. All simulations were run at constant temperature (300 K) and pressure (1 atm), using a Langevin damping coefficient of 0.5 fs^−1^ and a Berendsen thermostat relaxation time of τ_P_ = 0.1 ps. The particle-Mesh Ewald method was used to set the periodic boundary conditions that were used for long-range electrostatic interactions, and a real space cutoff of 10 Å was used. Conformations were sampled every 10 ps for subsequent analysis. All frames with time interval of 10 ps were saved to disk.

### MD analysis

Simulation trajectories were analyzed using the GROMACS 5.14 simulation package (66). For PCA, 3N∗3N atom covariance matrices of the protein displacement in simulations were generated based on backbone atoms (N, Cα, C, and O) of the *Pf*A-M17 crystal structure. PCs that taken together accounted for more than 50% of the overall covariance and were chosen for essential dynamics analysis. The GROMACS 5.14 simulation package was used to project the trajectory onto the top PCs. Graphs and plots were produced with GraphPad Prism, version 7.0. Molecular graphics were prepared with PyMOL 1.8.23 and VMD 1.9.3 (67).

## Data availability

The coordinates for the X-ray crystal structure of *Pf*A-M17_Inactive_ and *Pf*A-M17_Zn2+soak_ can be found at PDB IDs 7SRV and 7T3V, respectively.

## Supporting information

This article contains supporting information (16, 68).

## Conflict of interest

The authors declare that they have no conflicts of interest with the contents of this article.

## Supporting information

Supporting Material

SupMov6_2880x2160.10Mbps; VIDEO

Hex-M17; VIDEO

SupMov8_2880x2160.10Mbps; VIDEO

mono-M17; VIDEO
