# Supplementary material for: A metal ion–dependent conformational switch modulates activity of the Plasmodium M17 aminopeptidase
Source: J Biol Chem. 2022 Jun 9;298(7):102119. doi: 10.1016/j.jbc.2022.102119 (PMC9270245; doi:10.1016/j.jbc.2022.102119)
Supplement: Supporting Material [file mmc1.docx]

**Supporting Information**

**A metal ion-dependent conformational switch modulated the activity of the *Plasmodium* M17 aminopeptidase.**

Chaille T. Webb ^1^, Wei Yang^1a^, Blake T. Riley ^2^, Brooke K. Hayes^1^, Komagal Kannan Sivaraman^1^, Tess R. Malcolm ^1^, Stephen Harrop^3^, Sarah C. Atkinson ^2^, Itamar Kass^2,4,b^, Ashley M. Buckle^2^, Nyssa Drinkwater^1,*^, Sheena McGowan^1,*^

^1^ Biomedicine Discovery Institute, Department of Microbiology, Monash University, Clayton Melbourne, VIC 3800, Australia

^2^ Biomedicine Discovery Institute, Department of Biochemistry and Molecular Biology, Monash University, Clayton Melbourne, VIC 3800, Australia

^3^ Australian Synchrotron. 800 Blackburn Road, Clayton, VIC, 3168, Australia

^4^ Victorian Life Sciences Computation Center, Monash University, Clayton 3800, Victoria, Australia

*^a^ Current address Warshel Institute for Computational Biology, The Chinese University of Hong Kong, Shenzhen, 518172, China*

*^b^ Current address InterX LTD, Ramat-Gan, Israel.*

The following figures and movies are provided as supporting information:

**Supporting Information 1.** RMSD plot of monomeric and hexameric *Pf*A-M17 over course of molecular dynamics simulations.

**Supporting Information 2:** Radial distribution function (RDF) plot of water molecules in the zinc environment from the MD simulations of monomeric and hexameric *Pf*A-M17.

**Supporting Information 3:** RMSF analysis per residue throughout molecular dynamics simulations.

**Supporting Information 4:** Movie showing displacement along PC2 of *Pf*A-M17 hexamer simulation.

**Supporting Information 5:** Crystallographic Data Collection and Refinement Statistics.

**Supporting Information 6:** Movie showing morph between active and inactive *Pf*A-M17.

**Supporting Information 7:** X-ray fluorescence scan on *Pf*A-M17_Zn2+soak_ crystals.

**Supporting Information 8:** Alignment of the *Pf*A-M17_Active_ structure (PDB 3KQZ; blue) with *Pf*A-M17_Zn2+soak_ (aqua) structure.

**Supporting Information 9:** Analytical size exclusion chromatography of *wild type* and mutant *Pf*A-M17.

**Supporting Information 10:** Sequence alignment and sequence motif of L13 loop region from verified M17 aminopeptidases from a range of organisms.

**Supplementary References**

**Supporting Information 1:** RMSD plot of (A) monomeric, and (B) hexameric, *Pf*A-M17 over course of triplicate molecular dynamics simulations.


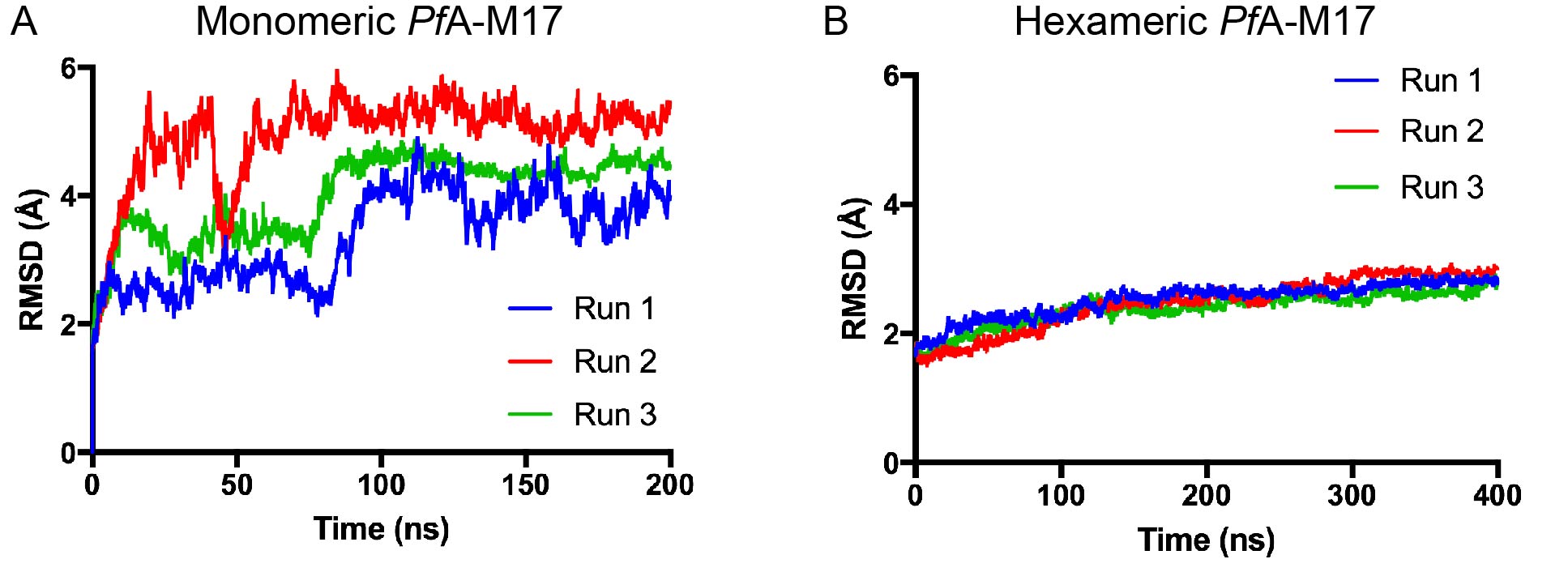


**Supporting Information 2:** **Radial distribution function (RDF) plot of water molecules in the zinc environment from the MD simulations of (A) monomeric *Pf*A-M17, and (B) hexameric *Pf*A-M17.** The RDF was calculated by the average of all six sites in the hexamer. Probability percentage was calculated by the occupancy of the water divided by the number of waters (as measured by oxygen atom of the waters) at any given distance to the zinc ions. The probability data were averaged from the six catalytic pockets of the hexameric *Pf*A-M17. The plots show that the catalytic water in simulation of monomer is unstable, however, the hexamer has a single water molecule in the catalytic position throughout the simulation. Corresponding movies depicting trajectories of monomeric *Pf*A-M17 (**C**), and hexameric *Pf*A-M17 (**D**) MD simulations centered on the active site metals and showing movement of water molecules. Active site zinc ions are shown as grey spheres, zinc coordinating residues are shown as stick (teal carbon atoms) and labelled, water molecules are shown as ball and stick and can move rapidly throughout movie.

**
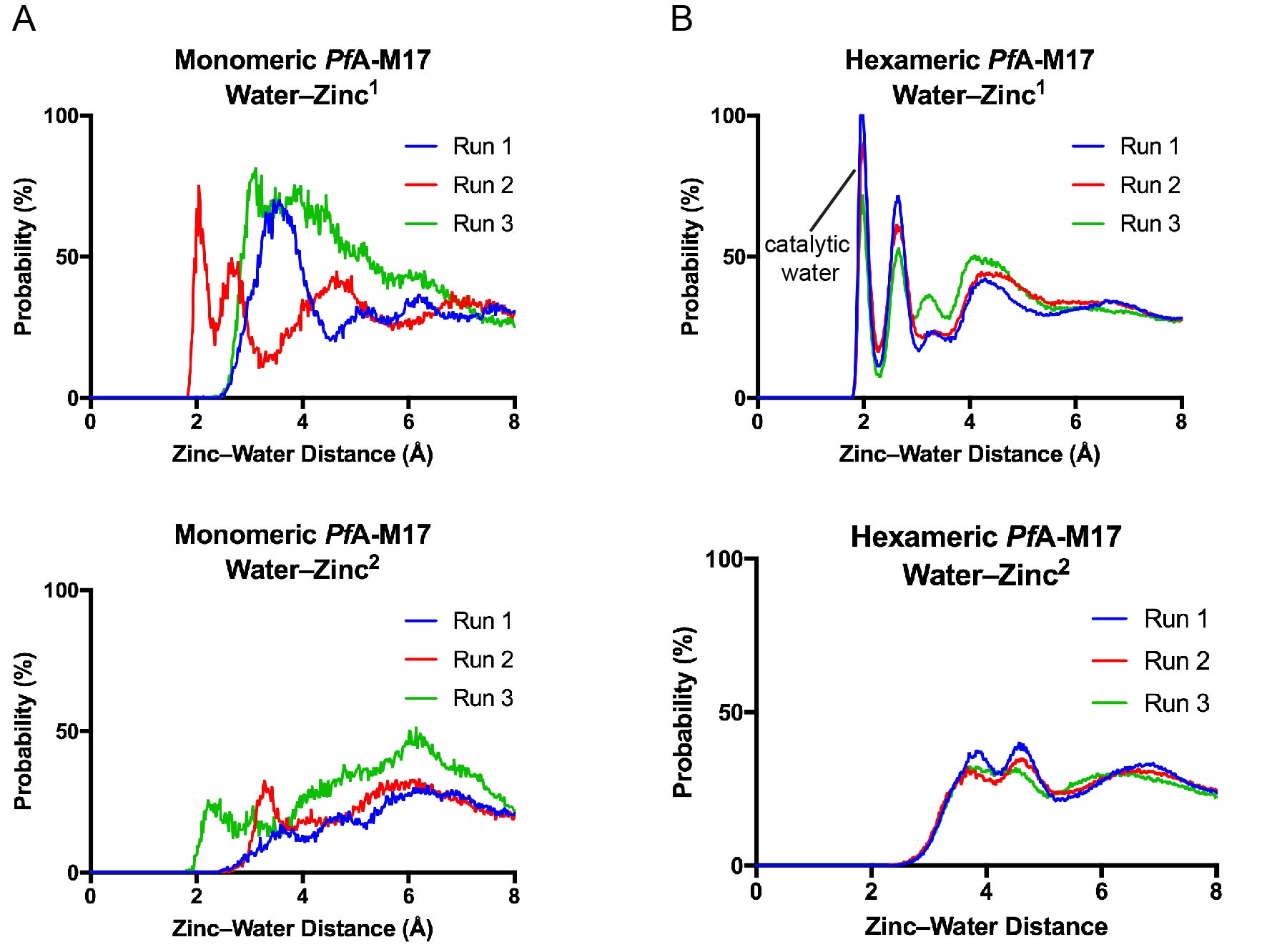
**

**C** <https://www.dropbox.com/s/ag4rjn9bqqkraor/mono-M17.mp4?dl=0>

**D** <https://www.dropbox.com/s/gh8n40wija293ry/Hex-M17.mp4?dl=0>

**Supporting Information 3:** **RMSF analysis per residue throughout molecular dynamics simulations.** (A) *Pf*A-M17 monomer throughout simulation. (B) RMSF analysis of the six individual chains in hexameric *Pf*A-M17 throughout simulation.


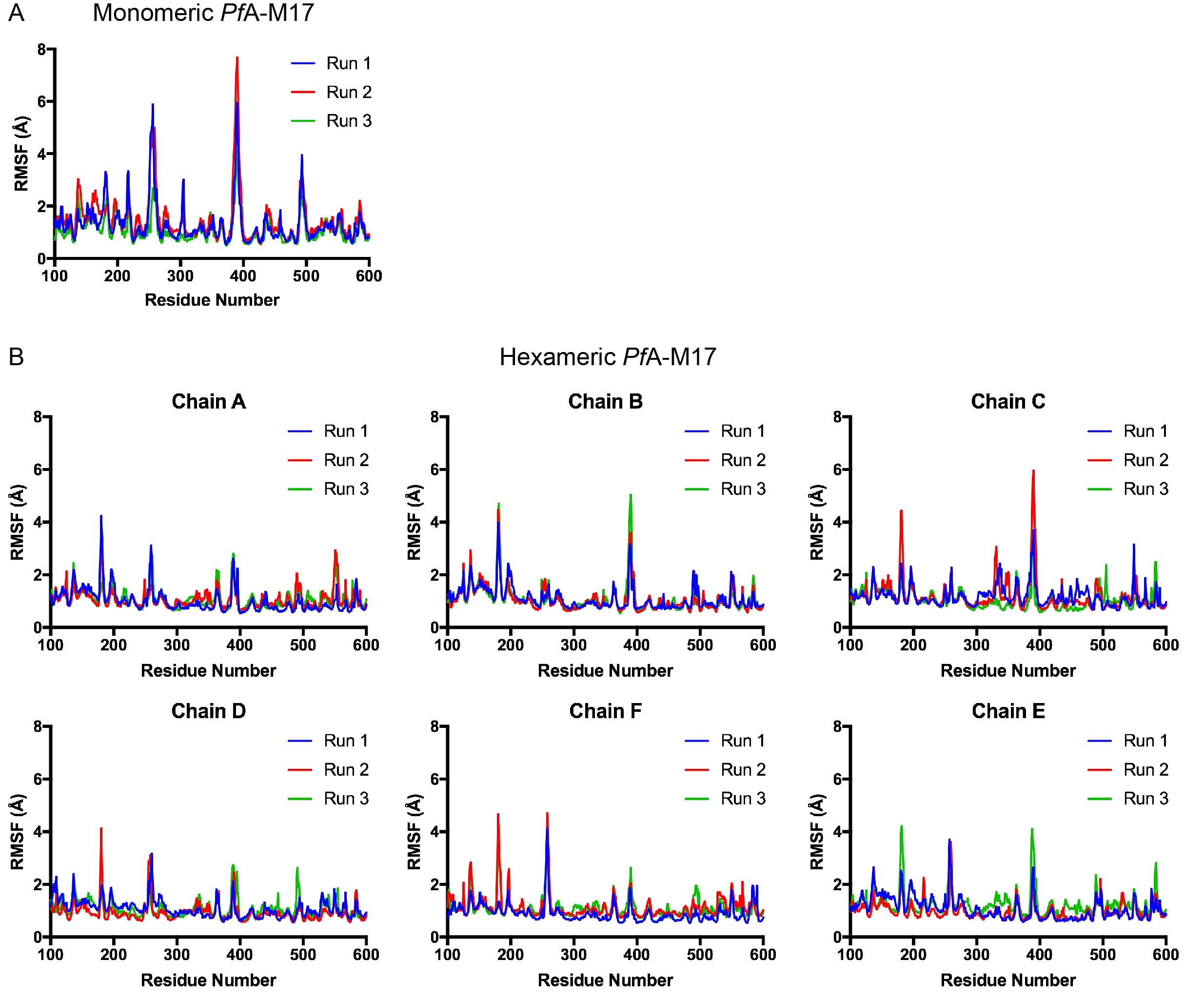


**Supporting Information 4:** **Movie showing displacement along PC2 of *Pf*A-M17 hexamer simulation.** *Pf*A-M17 trimer with greatest level of movement shown only (chains D, E, and F), with chain D in yellow, E in red, and F in orange. Where amino acids were not modelled in the crystal structures due to poor electron density, the main chain appears as dashes.

[https://www.dropbox.com/s/q6d2slta676kirg/SupMov6_2880x2160.10Mbps.mp4?dl=](https://www.dropbox.com/s/q6d2slta676kirg/SupMov6_2880x2160.10Mbps.mp4?dl=0)0

**Supporting Information 5: Crystallographic Data Collection and Refinement Statistics**

| **Dataset** | ***Pf*A-M17_Inactive_** | ***Pf*A-M17****_Zn2+soak_** | |
| --- | --- | --- | --- |
| PDB ID | 7SRV | 7T3V | |
| Condition | No Zn soak | + Zn soak | + Zn soak |
| *Data collection* | | | |
| Wavelength | 0.9537 | 0.9537 | 1.23 |
| Resolution range | 49.19 - 2.03 (2.103 - 2.03) | 46.92 - 2.303 (2.385 - 2.303) | 49.07 - 2.5 (2.589 - 2.5) |
| Space group | P 2 21 21 | P 2 21 21 | P 2 21 21 |
| Unit cell | 111.542 172.67 179.395 90 90 90 | 111.76 172.756 176.922 90 90 90 | 111.41 173.173 178.659  90 90 90 |
| Total reflections | 1626188 (82021) | 1398250 (135534) | 3548823 (142977) |
| Unique reflections | 233061 (11469) | 144872 (14858) | 119617 (11618) |
| Multiplicity | 7.0 (7.2) | 9.7 (9.1) | 29.7 (12.3) |
| Completeness (%) | 100 (100) | 95.48 (98.86) | 99.34 (93.96) |
| Mean I/sigma(I) | 7.1 (0.7) | 11.81 (1.26) | 8.50 (0.69) |
| Wilson B-factor | 36.52 | 44.06 | 64.35 |
| R-merge | 0.160 (2.748) | 0.1411 (1.56) | 3.19 (3.701) |
| R-meas | 0.188 (3.239) | 0.1489 (1.653) | 3.253 (3.861) |
| R-pim | 0.099 (1.696) | 0.04682 (0.5421) | 0.5837 (1.087) |
| CC1/2 | 0.997 (0.244) | 0.998 (0.743) | 0.0306 (0.306) |
| CC* |  | 1 (0.923) | 0.244 (0.684) |
| *Refinement statistics* | | | |
| Reflections used in refinement | 222828 (23032) | 144746 (14837) | |
| Reflections used for R-free | 11027 (1080) | 1993 (202) | |
| R-work | 0.1857 (0.3173) | 0.2200 (.39) | |
| R-free | 0.2146 (0.3562) | 0.2609 (0.4072) | |
| Number of non-hydrogen atoms | 25585 | 23508 | |
| macromolecules | 23559 | 23167 | |
| ligands | 128 | 90 | |
| solvent | 1898 | 279 | |
| Protein residues | 3082 | 3092 | |
| RMS(bonds) | 0.004 | 0.004 | |
| RMS(angles) | 0.68 | 0.60 | |
| Ramachandran favored (%) | 97.68 | 95.51 | |
| Ramachandran allowed (%) | 2.26 | 4.26 | |
| Ramachandran outliers (%) | 0.07 | 0.23 | |
| Rotamer outliers (%) | 1.00 | 3.15 | |
| Clashscore | 3.86 | 15.73 | |
| Average B-factor | 39.73 | 57.06 | |
| macromolecules | 39.34 | 57.10 | |
| ligands | 45.04 | 52.60 | |
| solvent | 44.11 | 53.71 | |

*Statistics for the highest-resolution shell are shown in parentheses*

**Supporting Information 6: Movie showing morph between active and inactive *Pf*A-M17.** Chain A in blue, B in purple, C in teal, D in yellow, E in red, and F in orange.

<https://www.dropbox.com/s/0t0hfn1g4ft5lf8/SupMov8_2880x2160.10Mbps.mp4?dl=0>

**Supporting Information 7:** X-ray fluorescence scan on *Pf*A-M17_Zn2+soak_ crystals performed on the MX2 (3ID1) beamline at the Australian Synchrotron.


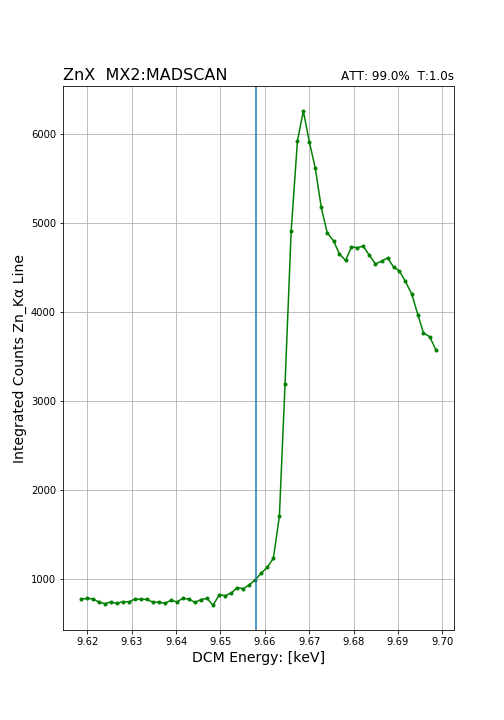


**Supporting Information 8. Alignment of the *Pf*A-M17_Active_ structure (PDB ID 3KQZ; blue) with *Pf*A-M17_Zn2+soak_ (PDB ID 7T3V; aqua).** By soaking the *Pf*A-M17_inactive crystals with zinc prior to data collection, the position of L13 loop returns to its position in the active form and coincides with the zinc atoms placed at sites 1 and 2 rather than sites 1 and 3. Alignment of L13 (residues D379-M392) from Chain A from both structures overlaps with a RMSD = 0.185, an alignment of the complete Chain A from both structures overlaps with a RMSD = 0.363.


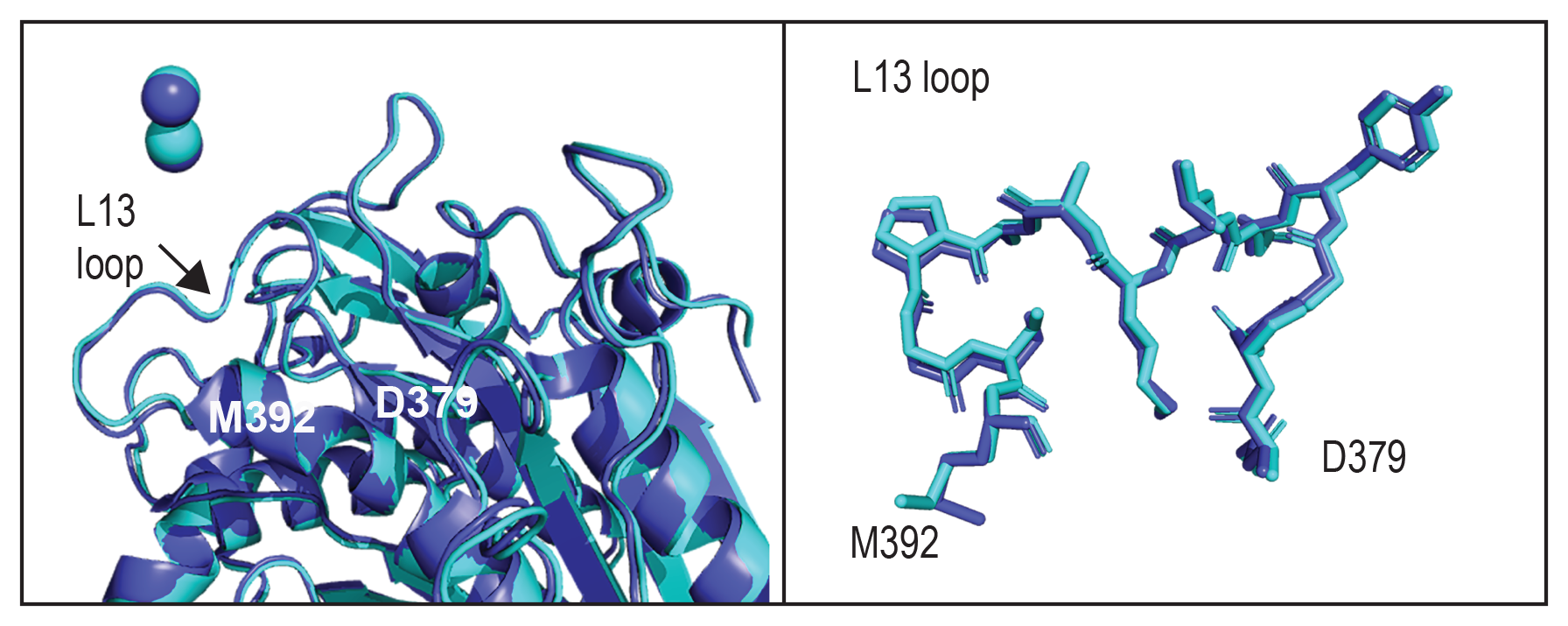


**Supporting Information 9: Analytical size exclusion chromatography of *wild type* and mutant *Pf*A-M17.** Absorbance traces of analytical gel filtration experiments to estimate oligomeric state of variant *Pf*A-M17 enzymes. Dashed lines indicate predicted elution volumes of different oligomeric states. Oligomeric state of *Pf*A-M17 (red, hexameric) and monomeric *Pf*A-M17(W525A+Y533A) (magenta, monomeric), confirmed by AUC (1). Based on interpolation of standard curve, elution volume of hexameric *Pf*A-M17 (red) corresponds to an approximate molecular weight of 330 kDa (molecular weight of hexameric *Pf*A-M17 calculated from amino acid sequence is 352 kDa), whereas elution volume of monomeric *Pf*A-M17(W525A+Y533A) (magenta) corresponds to approximate molecular weight of 68 kDa (monomeric *Pf*A-M17 molecular weight from amino acid sequence is 58.6 kDa). *Pf*A-M17(K386A) (orange) elution volume corresponds to an approximate molecular weight of 143 kDa, while *Pf*A-M17(D394A) (light blue), *Pf*A-M17(A387P) (dark blue), *Pf*A-M17(Δ388-389) (grey), and *Pf*A-M17(Δ388-390) (green) are all largely hexameric. The table depicts the calculated melting temperature, Tm, for *Pf*A-M17 proteins using differential scanning fluorimetry. The melting temperature is defined as the inflection point of the melting curve, corresponding to the peak of the first negative derivative of the curve.

|  | Tm (°C) |
| --- | --- |
| *Wild type Pf*A-M17 | 51.2 |
| *Pf*A-M17(K386) | 51.4 |
| *Pf*A-M17(D394A) | 49.0 |
| *Pf*A-M17(A387P) | 50.9 |


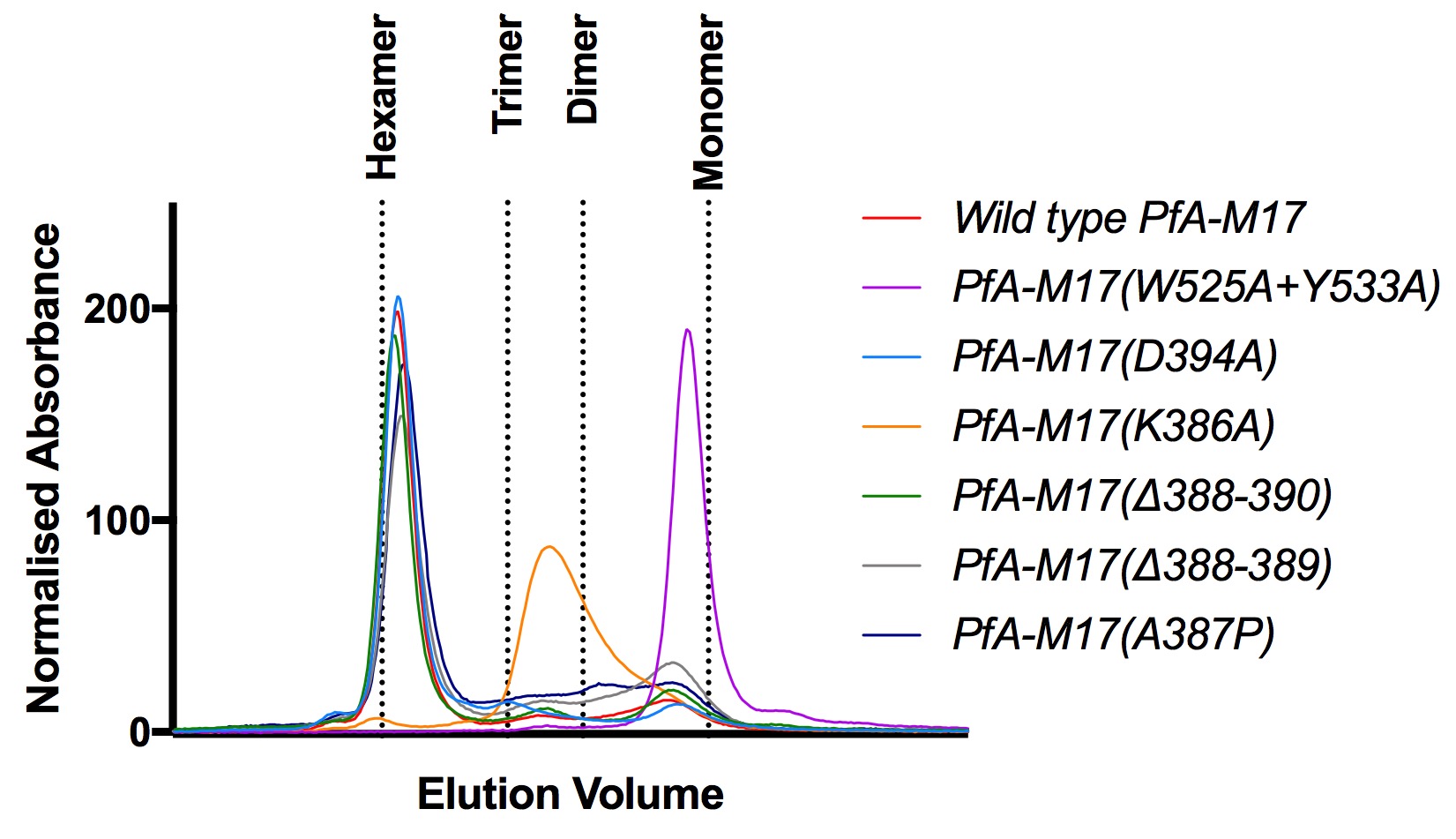


**Supporting Information 10: Sequence alignment and sequence motif of L13 loop region from verified M17 aminopeptidases from a range of organisms**. Conserved lysine residue (Lys386 in *Pf*A-M17) is highlighted in green, loop 13 residues in pink; yellow shows that Asp394 of *Pf*A-M17, representative of zinc binding site 3, and orange shows previously identified zinc binding residues Asp399 (metal site 2 and 3). **(A)** Asp394 (*Pf*A_M17 numbering) is conserved in M17 aminopeptidases from *Plasmodium* species that infect human and rodents (PlasmoDB references: (PF3D7_1446200;

PVX_118180; PKNH_1236000; PmUG01_12070800; PocGH01_12068600 and PBANKA_130990). **(B)** The amino acid frequency of each position in loop 13 (as per *Pf*A-M17 numbering) from 140 annotated M17 aminopeptidases from MEROPS clan MA, family M17. Shown is a graphical representation or ‘sequence logo’ where each stack is indicative for each position in the selected sequence. The height of the symbols is indicative of the relative frequency of each amino acid at that position. (2) **(C)** Selected key representatives from MEROPS annotated collection to show diversity between M17 aminopeptidases from different species and kingdoms.

**
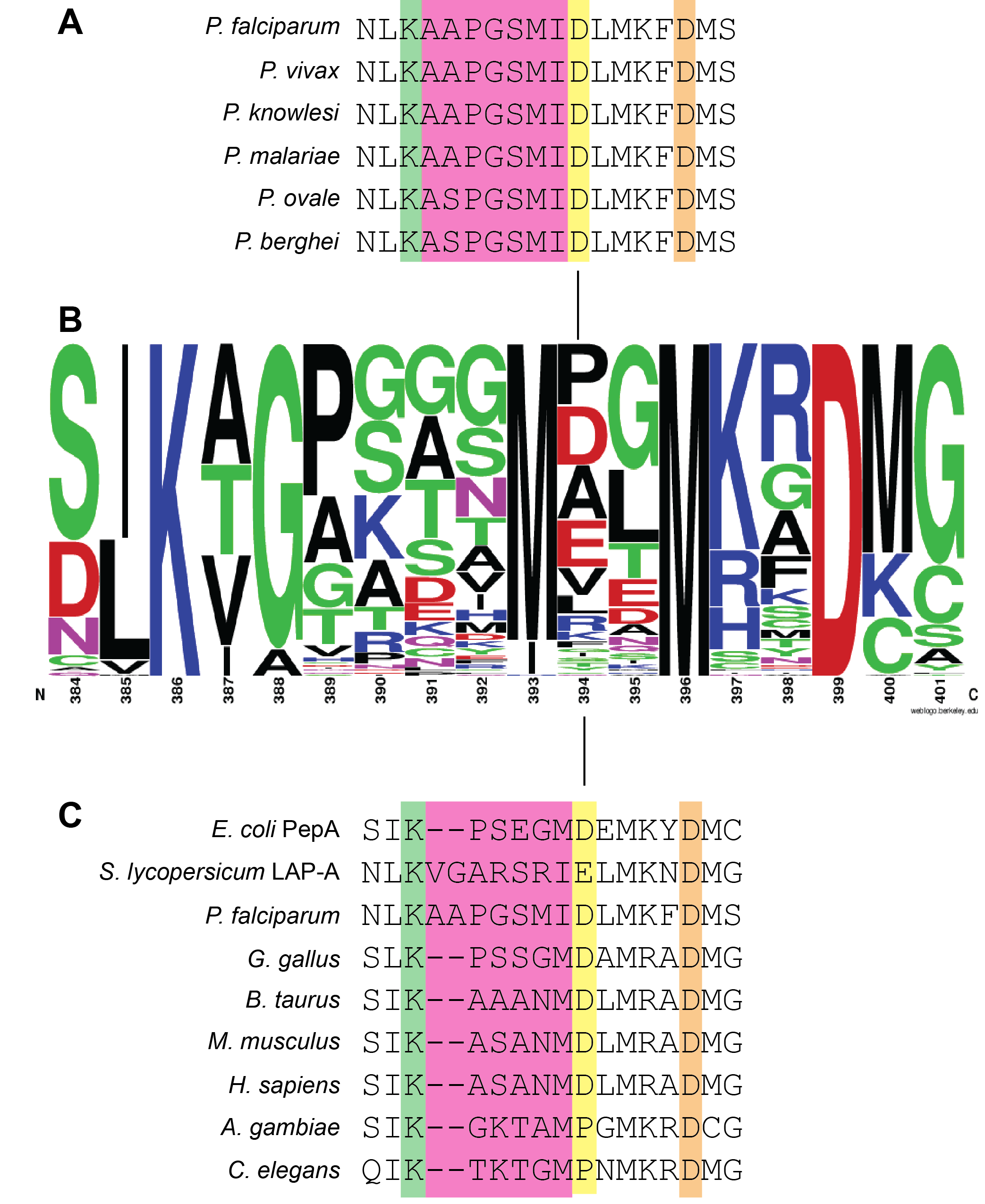
**

**Supporting References**

1. T. R. Malcolm *et al.*, Active site metals mediate an oligomeric equilibrium in Plasmodium M17 aminopeptidases. *J Biol Chem* **296**, 100173 (2021).

2. G. E. Crooks, G. Hon, J. M. Chandonia, S. E. Brenner, WebLogo: a sequence logo generator. *Genome Res* **14**, 1188-1190 (2004).
